# Essential function of adaptor protein Nck1 in platelet-derived growth factor receptor signaling in human lens epithelial cells

**DOI:** 10.1038/s41598-022-05183-1

**Published:** 2022-01-20

**Authors:** Pussadee Paensuwan, Jatuporn Ngoenkam, Apirath Wangteeraprasert, Sutatip Pongcharoen

**Affiliations:** 1 Department of Optometry, Faculty of Allied Health Sciences, Naresuan University, Tapho District, Phitsanulok, 65000 Thailand; 2 Department of Microbiology and Parasitology, Faculty of Medical Science, Naresuan University, Tapho District, Phitsanulok, 65000 Thailand; 3 Department of Medicine, Faculty of Medicine, Naresuan University, Tapho District, Phitsanulok, 65000 Thailand

**Keywords:** Mechanisms of disease, Medical research

## Abstract

Binding of platelet-derived growth factor-BB (PDGF-BB) to its cognate receptor (PDGFR) promotes lens epithelial cell (LEC) proliferation and migration. After cataract surgery, these LEC behaviors have been proposed as an influential cause of posterior capsule opacification (PCO). Stimulated PDFGR undergoes dimerization and tyrosine phosphorylation providing docking sites for a SH2-domain-containing noncatalytic region of tyrosine kinase (Nck). Nck is an adaptor protein acting as a linker of the proximal and downstream signaling events. However, the functions of Nck1 protein in LEC have not been investigated so far. We reported here a crucial role of Nck1 protein in regulating PDGFR-mediated LEC activation using LEC with a silenced expression of Nck1 protein. The knockdown of Nck1 suppressed PDGF-BB-stimulated LEC proliferation and migration and disrupted the cell cycle progression especially G1/S transition. LEC lacking Nck1 protein failed to exhibit actin polymerization and membrane protrusions. The downregulation of Nck1 protein in LEC impaired PDGFR‐induced phosphorylation of intracellular signaling proteins, including Erk1/2, Akt, CREB and ATF1, which resulted in inhibition of LEC responses. Therefore, these data suggest that the loss of Nck1 expression may disturb LEC activation and Nck1 may potentially be a drug target to prevent PCO and lens-related disease.

## Introduction

Anterior lens epithelial cells (LEC) proliferate and migrate to the lens equator and then differentiate into lens fiber cells during embryonic development^1^. However, abnormal growth of LEC can be pathological^2^. Physiological change of aging human lens leads to lens opacities^3,4^. An aged-related lens degeneration disorder, known as cataract, is a major cause of vision loss and blindness^4^. One of the complications associated with modern cataract surgery, phacoemulsification cataract surgery with artificial foldable intraocular lens (IOL) implantation, is a posterior capsule opacification (PCO). The prevalence of PCO is up to 34.3% in adults and almost 100% in children^5,6^. The PCO development is a very dynamic process involving proliferation, migration and epithelial-mesenchymal transition (EMT) of residual LECs^7^. It is a wound-healing response of the remaining LECs in the anterior lens capsule bag leading to thickness and opacity of the lens. The residual of anterior LEC undergo proliferation and migration into the posterior lens capsule to form elschnig’s pearls and fibrotic change, resulting in visual acuity impairment when the visual axis is involved^2,6,8^. Mechanical and pharmaceutical methods have been established for PCO prevention, focusing on inhibiting or removing of the residue LEC^9,10^. Nevertheless, mechanisms of therapeutic strategies underlying such PCO prevention remain unclear.

Many growth factors are found in ocular media, especially aqueous humor, which regulate lens cell behaviors^11^. Platelet-derived growth factor (PDGF) is one of the growth factors demonstrated to be a mitogen that regulates LEC proliferation and migration^1,12,13^. PDGF family encodes four genes that form five active dimeric isoforms: PDGF-AA, PDGF-AB, PDGF-BB, PDGF-CC and PDGF-DD^14^. PDGF-BB is the only Food and Drug Administration (FDA)-approved growth factor successfully and safely used for chronic wound healing treatment^15,16^^.^ PDGF is highly expressed in the iris and ciliary body, whereas its cognate receptor tyrosine kinase (RTK), called platelet-derived growth factor receptor (PDGFR), is expressed on the LEC surface at approximately 35,000 PDGF-BB binding sites^17^. Upon ligand engagement, PDFGR, which has an intrinsic protein tyrosine kinase activity, undergoes dimerization and auto-tyrosine phosphorylation creating docking sites for numerous Src homology 2 (SH2)-domain-containing cytosolic proteins^18–20^. Of these, an adaptor protein called the non-catalytic region of tyrosine kinase (Nck) is recruited to the phosphorylated tyrosine residues of PDGFR to initiate downstream signaling pathways promoting cell growth^21,22^.

Nck is a widely expressed adaptor protein composed of one SH2-domain and three SH3 domains (SH3.1, SH3.2 and SH3.3)^23^. Nck plays a pivotal role in actin reorganization, cell movement and cell adhesion^24^. Nck has two highly related members, Nck1 (Nckα) and Nck2 (Nckβ), which share 68% amino acid identity^23^. However, the non-redundant function of both Nck isoforms in humans has been shown in which Nck1, rather than Nck2, plays a significant role in CD3ε-mediated T cell activation^25,26^. After PDGFR ligation, receptor signaling is transmitted to activate several intracellular signaling cascades, including the mitogen-activated protein kinase (MAPK) and the phosphatidylinositol 3-kinase-Akt (PI3K-Akt) pathways. It has been shown that Nck protein acts as a linker to recruit and activate other signaling proteins and transmembrane receptors in multiple intracellular pathways^27^. Nevertheless, the relation in cellular mechanism between PDGFR and Nck in LEC has not been established. It has been revealed that Nck1 downregulation impairs several downstream signaling cascades^21,25,26^. Moreover, Nck1 is essential for regulating actin polymerization^28^. From these findings, Nck1 adaptor protein related with PDGFR signaling might be the target to open new opportunities to prevent lens-related disease.

Since the role of Nck1 adaptor protein might be involved in the function of PDGFR in human LEC, this study was aimed at investigating Nck1 functions on the proliferation, migration, actin polymerization, and intracellular signal transduction pathways in PDGFR-mediated LEC. With Nck1 knockdown LEC, our work provides evidence that Nck1 protein might be an essential adaptor in regulating cellular outcomes of LEC.

## Results and discussion

Given that unusual LEC growth and differentiation could cause worsen pathological conditions, research findings targeting critical proteins such as PDGF in LEC may open new opportunities for designing therapeutic strategies to improve visual impairment in patients with lens-related diseases^29–32^. Many proteins are involved in PDGFR-mediated signaling, one of them being Nck adaptor protein. It has been shown that Nck can be recruited to bind the PDGFR via its SH2 domain^21,33,34^. However, the functional role of Nck1 proteins in PDGFR-induced LEC is not well understood. To this end, Nck1 functions were investigated using LEC with a silenced expression of Nck1 protein.

**Figure 1:**
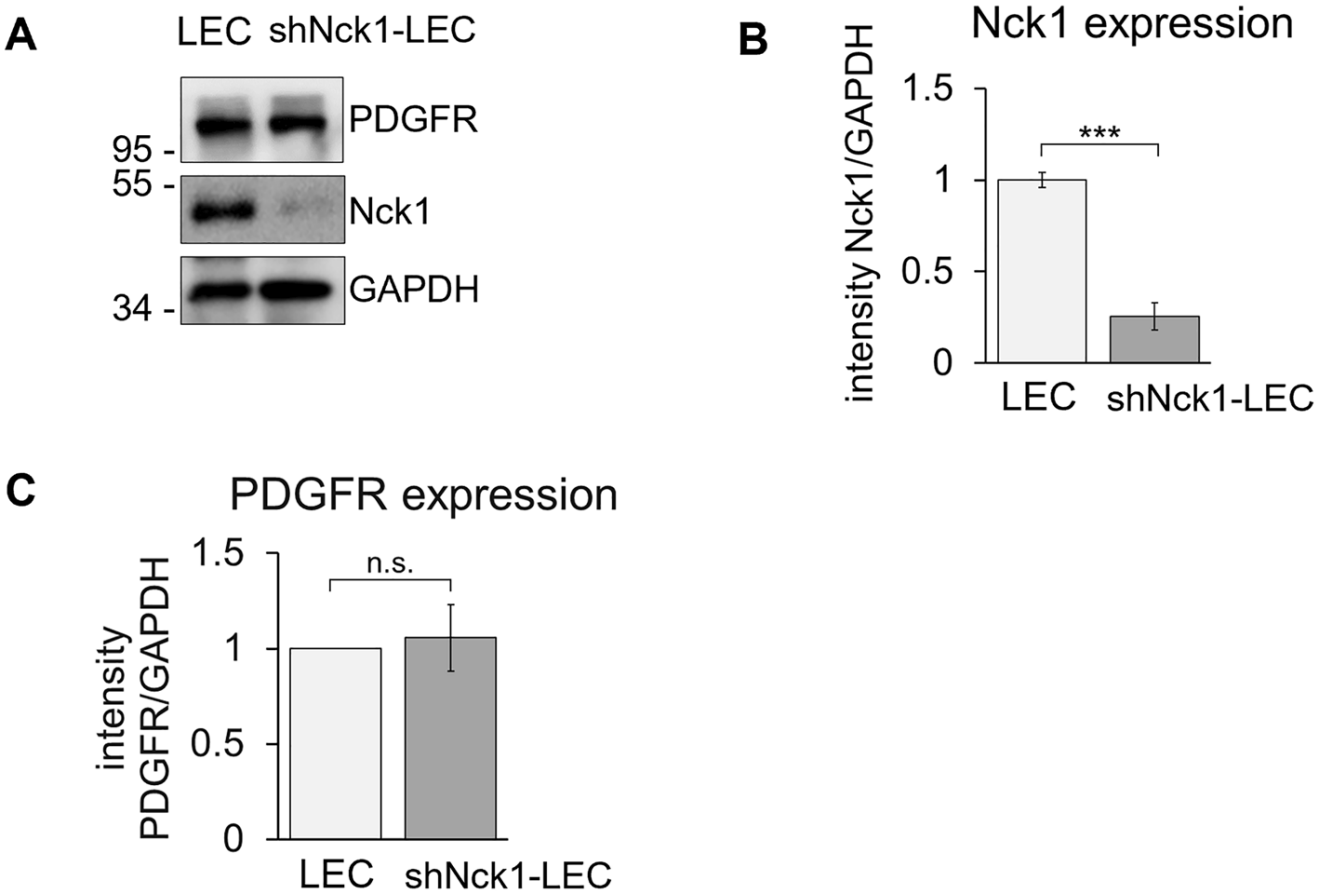
Expression of Nck1 adaptor protein was reduced after transfection with Nck1- shRNA expression vector. (**A**) LEC were transfected with a Nck1-specific shRNA expression vector. Following 48 h of transfection, a stable clone of Nck1 knockdown LEC was selected in medium containing 4 µg/ml puromycin. Cell extracts were subjected to western blot analysis probing with anti-Nck1, anti-PDGFR and anti-GAPDH antibodies. (**B**) Values in (**A**), quantification of signal intensity, presented as the ratio of Nck1 or PDGFR to corresponding GAPDH in relative to the protein of choices expressed in control LEC. A clone with a similar level of PDGFR expression were selected for further studies. Data are representative of three experiments (mean ± SEM). n.s. = not significant, ***p < 0.001.

Western blot analysis revealed that the Nck1-shRNA-transfected cells markedly decreased the expression of Nck1 protein (*p* < 0.001; Fig. 1A), which was named as shNck1-LEC. The result showed the reduction of Nck1 protein by approximately 80% compared with control LEC (Fig. 1B). Knockdown of Nck1 in LEC did not affect to PDGFR expression as it showed a similar PDGFR expression as the control cells (Fig. 1C) and was used for further studies.

### Knockdown of Nck1 protein inhibits PDGF-BB-induced LEC proliferation

**Figure 2:**
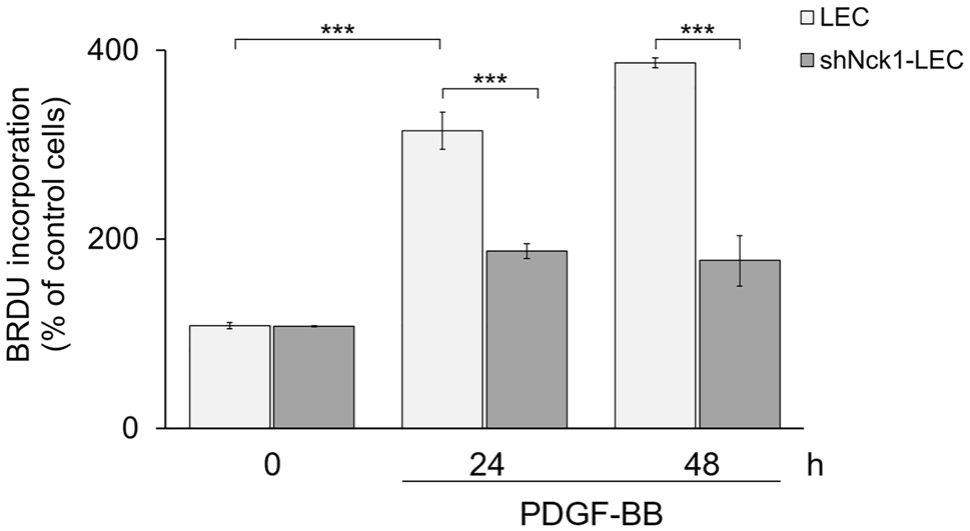
Knockdown of Nck1 adaptor protein suppresses PDGF-BB-induced LEC proliferation. LEC and shNck1 LEC were either stimulated with 30 ng/ml PDGF-BB or left untreated at 37 °C for 24 and 48 h. BrdU was added to the cells at a final concentration of 10 μM. Cell proliferation was examined using ELISA-based BrdU incorporation assay. Data are representative of four independent experiments and represent the mean ± SEM. ***p < 0.001.

As we showed that LEC and shNck1-LEC expressed the same level of PDGFR expression, the functional role of Nck1 adaptor protein in both cells was assessed in response to recombinant human platelet-derived growth factor-BB (PDGF-BB) stimulation. As expected, PDGF-BB-stimulated LEC showed markedly an increased cell proliferation compared with the unstimulated cells (*p* < 0.001) (Fig. 2). Thus, PDGF-BB is a potent growth factor that could modulate PDGFR signaling eliciting LEC growth. Next, the role of Nck1 protein in LEC proliferation was examined. The increase of cell proliferation was still found in shNck1-LEC; however, this proliferation rate was significantly lower than the PDGF-BB-stimulated LEC after 24 and 48 h (*p* < 0.001) (Fig. 2). These suggested that the knockdown of Nck1 significantly decreased PDGF-BB-induced LEC proliferation.

### Nck1 adaptor protein regulates cell cycle progression of LEC

**Figure 3:**
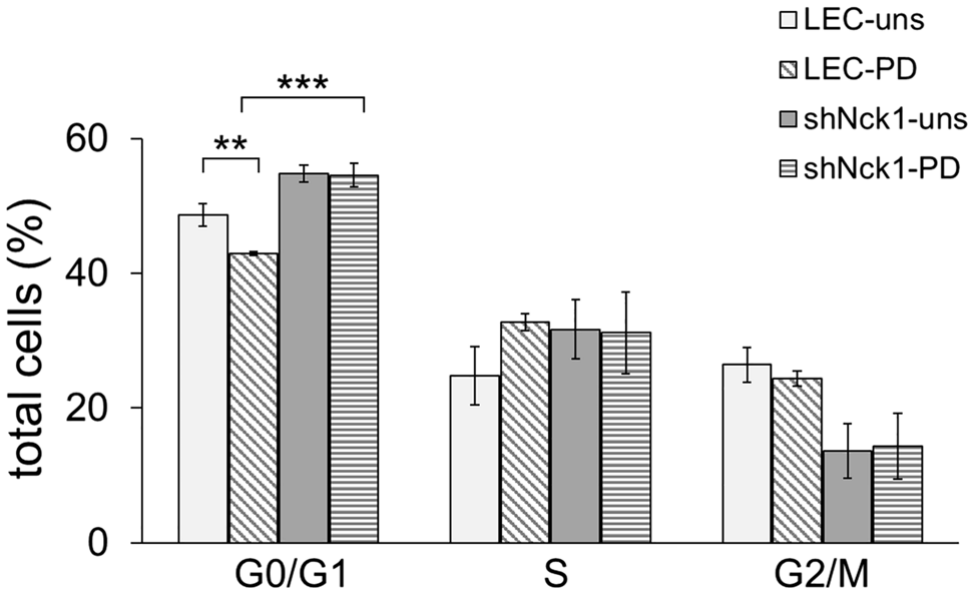
Nck1 knockdown disturbs cell cycle progression of LEC. Flow cytometry was used to detect cell cycle distribution. After 24 h treatment with 30 ng/ml PDGF-BB, a propidium iodide (PI)-stained cells were analyzed for the percentage of cell population at each phase of the cell cycle. The percentages of cells in G1, S, and G2/M is shown in graph representing the mean ± SEM. Data are representative of three independent experiments. **p < 0.01, ***p < 0.001. uns = unstimulated cells, PD = PDGF-BB-stimulated cells.

Since Nck1 protein depletion caused the suppression of LEC proliferation, the cell cycle regulation might be impaired, causing cell cycle arrest. To explore this notion, the effect of Nck1 reduction on the cell cycle progression of LEC was investigated by measuring propidium iodide (PI)-stained DNA content. After stimulation with PDGF-BB, LEC showed a significant decrease in the percentage of cell number in G0/G1 phase (p < 0.01), while the percentage of cells in S phase increased compared with the unstimulated LEC (Fig. 3). This result confirmed that PDGF-BB is an efficient growth factor contributing to cell cycle transition from G1 to S phase via PDGFR-mediated signaling^35,36^. However, in the absence of growth factors or impaired growth factor receptor-mediated signaling, it appears that cells are unable to promote the progression in G1 and the G1/S transition is inhibited^37^. This is consistent with our results in which shNck1-LEC showed the switch from proliferation to cell cycle arrest following PDGF-BB treatment. The Nck1 knockdown induced cell cycle arrest at G0/G1 phase as seen by an increased percentage of cell proportion in G0/G1 phase (*p* < 0.001) (Fig. 3). This suggested that the reduction of Nck1 adaptor protein leads to a disruption of the cell cycle progression especially G1/S transition. Therefore, our present data supported the notion that the knockdown of Nck1 adaptor protein expression can induce cell cycle arrest at G0/G1 leading to the prevention of LEC proliferation.

### Knockdown of Nck1 inhibits PDGF-BB-induced LEC migration

Migration of LEC is a dynamic process involving multiple LEC cellular events^7,8^. It has also been reported that LEC migration is a crucial pathological mechanism of cataract surgery-related complications, known as the PCO. The development of PCO is caused by the residual of LECs, in response to surgery, that migrate away from the anterior into the posterior lens capsule leading to recurrent vision loss^2,6,8^. Thus, inhibition of LEC migration might be useful for preventing PCO development. Since cell migration is sensitive to the proliferative state, the nonproliferating cells would not undergo migration. Moreover, our studies showed that signaling through Nck1 protein regulated both proliferation and cell cycle arrest. We thus hypothesized that Nck1 knockdown might suppress migration of LEC. Following this, the role of Nck1 on LEC migration was assessed using a conventional scratch wound healing assay.

**Figure 4:**
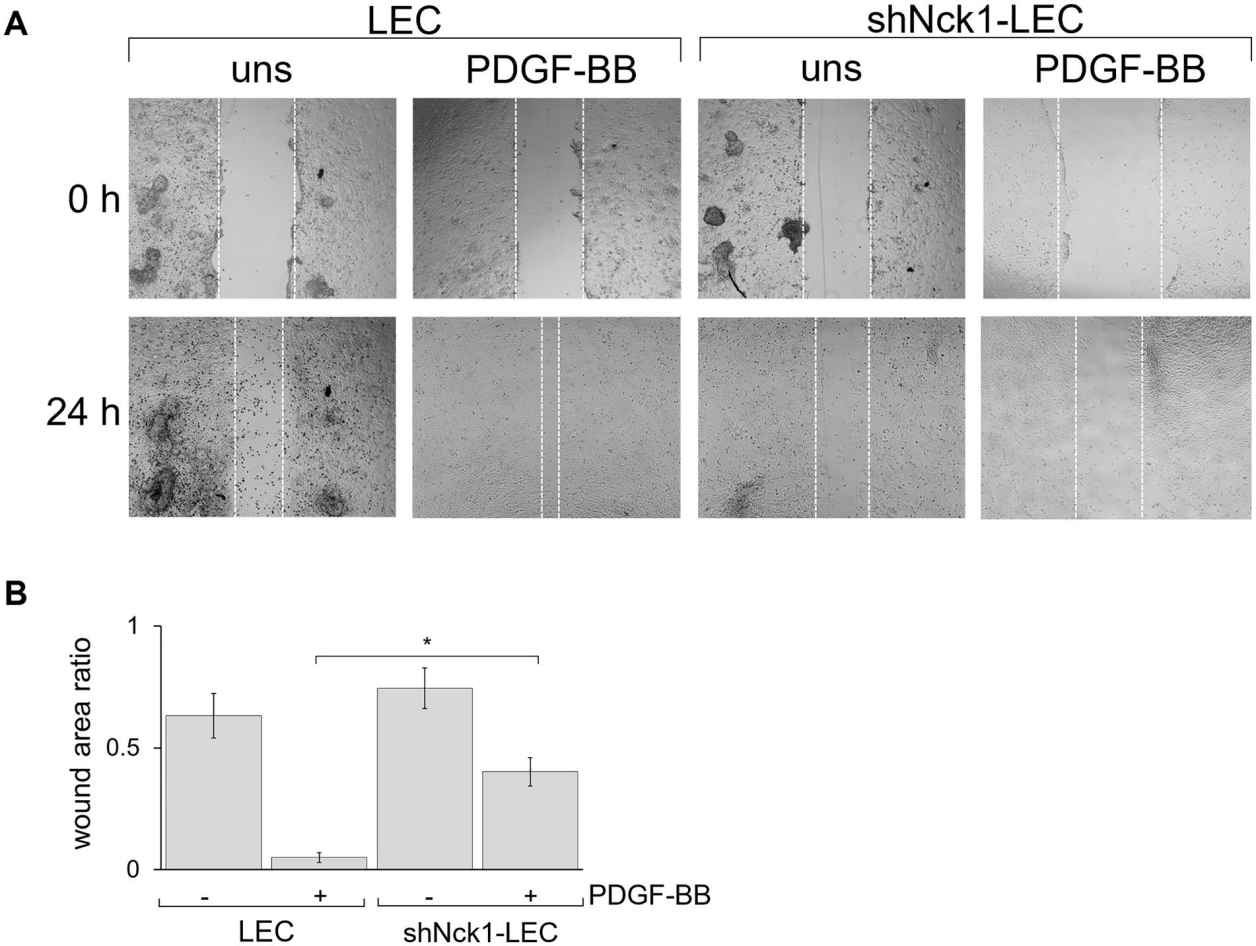
Knockdown of Nck1 inhibits PDGF-BB-induced LEC migration. A scratched wound was created in the absence or presence of 30 ng/ml PDGF-BB. The covered wound area quantification was acquired at 24 h. (**A**) Representative images of wound closure of LEC at 0 and 24 h. (**B**) Quantification of the wound area ratio is shown in graph representing the mean ± SEM. Data are representative of three independent experiments. **p < 0.01.

As shown in Fig. 4, PDGF-BB treatment significantly accelerated LEC migration, resulting in a substantial decrease of wound area as compared to the unstimulated cells (*p* < 0.01, Fig. 4). However, the decrease of wound area was markedly delayed in shNck1-LEC. The shNck1-LEC showed a significantly wider wound area than LEC. One possibility is that PDGFR-associated activation required Nck1 protein to link the signaling from receptor regulating cell migration via p21-activated kinases (Pak) activation. The small GTPases Rac and CDC42 are activated after PDGFR engagement, and subsequently, contribute to the interaction between Nck and Pak, promoting cell migration^38^. Moreover, this cell migration impairment is in agreement with the present study demonstrating the occurrence of cell cycle arrest in shNck1-LEC. It is likely that the growth-arrested cells did not migrate in response to PDGF-BB, as previously reported that cells arrested in G0/G1 contribute to the inhibition of the cell migration and invasion^39^. It has been revealed that the lack of Nck protein interrupts chemotactic response to PDGF by interfering the interaction of Nck and Crk-associated substrate (p130^Cas^), which is implicated in cell migration^40^. Together, these results established that Nck1 adaptor protein is required for LEC migration via PDGFR-mediated signaling. Collectively, our results confirmed that the depletion of Nck1 expression suppressed cell proliferation and cell migration and induced cell cycle arrest in LEC.

### Nck1 adaptor protein is required for a sustained cell shape and actin reorganization

Actin reorganization is essential for cellular movement, change in the cell shape, and transportation of vesicles^24,41^. Since the cellular processes of PCO involved actin cytoskeleton dynamics, interfering actin polymerization would be a target for PCO inhibition^42^. However, the mechanism of disruption of actin polymerization or reorganization for PCO prevention remains unclear. Regulation of actin remodeling requires tyrosine phosphorylation to create the docking sites for SH2 domain-containing protein recognition^43^. It has been shown that SH2-domain-containing Nck proteins are recruited to bind receptor tyrosine kinases (RTKs), PDGFR, connecting to actin cytoskeleton regulators. An earlier finding has confirmed the involvement of Nck-PDGFR interaction in actin re-arrangement after PDGFR triggering^28^. Thus, Nck adaptor protein acts as a scaffold protein that recruits various signaling proteins to induce actin polymerization^23,44^. From the above findings, the Nck-PDGFR interaction may be directly or indirectly involved in actin cytoskeleton dynamics in LEC. However, little is known about the mechanism of this interaction in regulating the actin polymerization towards the PCO formation. To determine this, shNck1-LEC was used as a model to characterize cellular morphological change and the actin rearrangement associated with Nck1 function in LEC.

**Figure 5:**
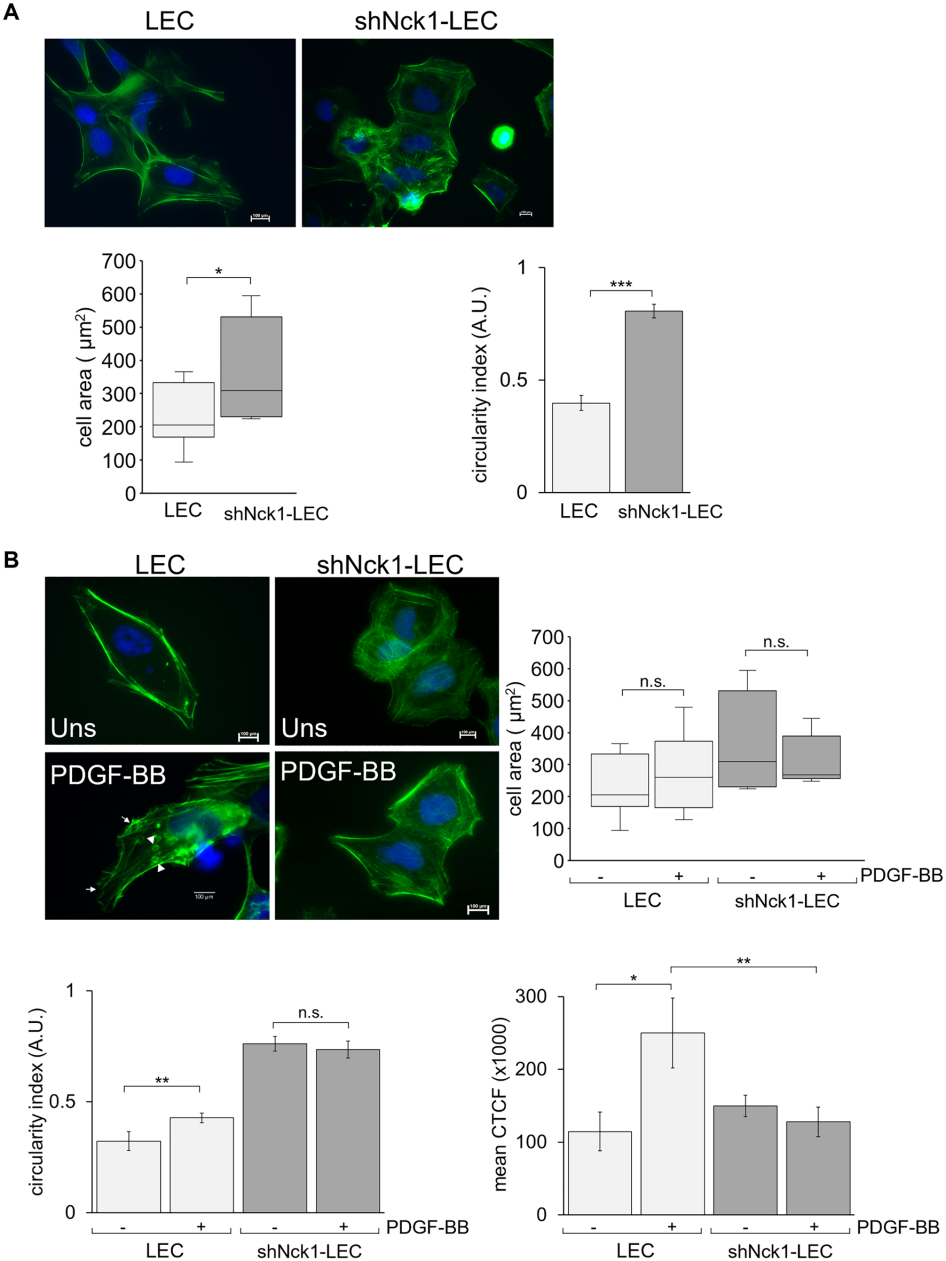
Nck1 adaptor protein is required for a sustained cell shape and actin reorganization. Cells were stained for actin filaments with AlexFluor 488‐phalloidin staining (green). Green fluorescence signals were observed with 600× magnification. (**A**) Nck1 knockdown changed cell morphology. Quantification of areas and perimeters of at least 10 cells from three separate experiments were measured. Graph illustrates cell area and cell circularity. (**B**) Upon PDGF-BB stimulation, peripheral and dorsal ruffling were induced as detected by staining for F-actin. Cell area, cell circularity and corrected total fluorescence (CTCF) intensity were quantified. Data are representative of three independent experiments and represent the mean ± SEM. Arrows and arrow heads indicate peripheral and dorsal ruffling, respectively. n.s. = not significant, *p < 0.05, **p < 0.01.

Under microscopic observation, in resting state LEC had an elongated and polar shape. In contrast, reduced Nck1 expression affected the cell shape of LEC, evidenced by the appearance of a more extensive and rounder phenotype than the control cells. This suggested that LEC with Nck1 downregulation could not sustain a regular shape. Consistent with the image analysis, shNck1-LEC exhibited a significant increase in cell area and circularity index indicating more prominent and rounder cells than control cells (Fig. 5A). Moreover, LEC also exhibited a morphological change with membrane protrusions formation, including peripheral and dorsal ruffling, after PDGF-BB treatment (Fig. 5B). The dorsal ruffles were found colocalized with the actin-rich ring at apical surfaces of cells, whereas the peripheral ruffles were observed as protrusions of the leading edge with densely actin branches at the cell periphery^45^. Interestingly, in contrast to LEC, this shape change did not occur in shNck1-LEC following PDGF-BB stimulation. In shNck1-LEC, the membrane ruffles formation was abolished (Fig. 5B). This result was likely to be associated with the impaired migration of shNck1-LEC, as the protrusive leading edges are a characteristic of migrating cells in response to PDGF^46^. However, cell area did not change after cell stimulation both in LEC and shNck1-LEC as compared to unstimulated cells.

Next, the relevance of Nck1 adaptor protein in the actin cytoskeleton was investigated. LEC exhibited a significant visible actin stress fiber, being aligned with the long axis of the cell in PDGF-BB-stimulated LEC than unstimulated LEC (*p* < 0.05) (Fig. 5B). However, short actin fibrils were abundantly distributed throughout in shNck1 LEC. There was a significant decrease of polymerized actin level in stimulated shNck1-LEC compared with the stimulated LEC (*p* < 0.01), suggesting that there was a considerable loss of actin polymerization (F‐actin) in shNck1-LEC. The formation of the membrane protrusions has been shown to be driven by the downstream signaling of PDGFR via the Rho GTPases activation. The explanation for these cellular behaviors might be that Nck1 protein is the downstream-associated adaptor of Rho GTPases activation in particularly RAC protein^47^. Rac-deficient cells exhibit impaired lamellipodium extension, membrane ruffling and migration in multiple cell types^48^. Our result showed that the failure of organized stress fibers occurred in the shNck1-LEC. Another possible explanation for this result is that the reduction of Nck1 protein might lead to the lack of Nck1 mediators forming multi-protein signaling complexes to the actin polymerization machinery. It has been shown that the Neural Wiskott-Aldrich syndrome protein (N-WASp) and actin-related protein 2/3 (Arp 2/3) are activated creating actin assembly after PDGFR triggering. Nck can bind the tyrosine-phosphorylated PDGFR via its SH2 domain while using its SH3 domain to bind the proline-rich sequence (PRS) of N-WASp. Moreover, The C-terminus of N-WASp binds constitutively to the Arp2/3 complex and dramatically stimulates actin filament nucleation and dorsal ruffling^23,49^. It is reasonable to suggest that Nck protein provides a link between PDGFR and N-WASp to regulate the actin polymerization.

Thus, Nck functions as a mediator of tyrosine-phosphorylated proteins and PRS-bearing proteins, through its SH2 and SH3 domains, respectively. Furthermore, Nck also acts as a linker to recruit and activate other proteins or transmembrane receptors in multiple intracellular pathways^27^. For this reason, Nck is classified as an “adaptor” protein that consequently engages the proximal signaling pathway through its SH2 domain and the downstream signaling pathway through its SH3 domains^50^. These observations indicated a vital role of Nck1 adaptor protein in the PDGFR signaling and actin cytoskeleton of LEC.

### Nck1 regulates the activation of the PDGFR-mediated signaling pathway

Upon ligand engagement, PDFGR undergoes dimerization and auto-tyrosine phosphorylation, providing an anchor site for SH2-domain-containing Nck proteins^19,20^. It has been shown that Nck1 protein is recruited to bind phospho-Tyr-751 in the PDGFβ receptor^21^. To demonstrate the influence of the Nck1 downregulation in signal transduction of LEC, downstream signaling proteins of PDGFR were also assessed.

**Figure 6:**
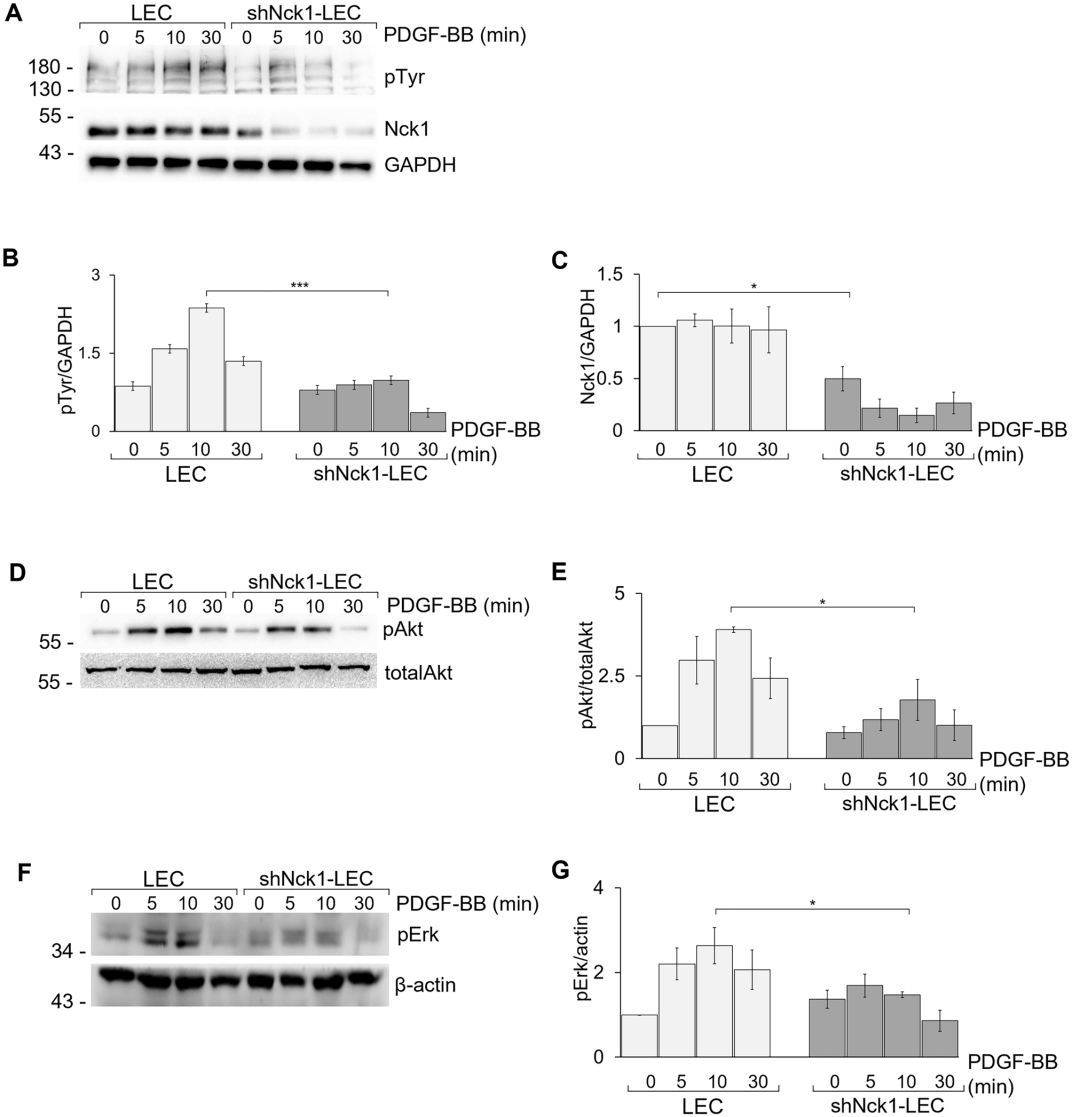
Nck1 knockdown impaired PDGFR-mediated signaling pathway. LEC and shNck1 LEC were either stimulated with 30 ng/ml PDGF-BB or left untreated at 37 °C for the indicated times. Cell lysates were separated by SDS-PAGE and the western blot was probed with (**A**, **B**) anti-phospho-tyrosine (p-Tyr, 4G10), (**A**, **C**) anti-Nck1, (**D**, **E**) anti-phospho-Akt (pAkt) and (**E**, **F**) anti-phospho-Erk1/2 (pErk1/2) antibodies. The quantified signal intensities are presented as a ratio of the protein of choice to the corresponding GAPDH values normalized to the value of unstimulated LEC. Data are representative of three experiments (mean ± SEM). *p < 0.05, **p < 0.01.

As seen in Fig. 6A, the result showed an increase of tyrosine phosphorylation (pTyr) at a band of approximately 180 kDa, which is similar to the size of PDGFR. This tyrosine phosphorylation was clearly detected within 10 min after stimulation. Interestingly, a significant decrease of level of tyrosine phosphorylation was found in shNck1-LEC (Fig. 6B,C). One possible explanation is that Nck1 protein might be involved in the recruitment of kinases to the PDGFR. This is consistent with a previous study showing that Nck binds indirectly to the PDGFR through p130^cas^, a downstream molecule of c‐Src kinase^40^. Another possibility is that the actin rearrangement is essentially required for sustained PDGFR phosphorylation that is associated with the requirement of Nck1 protein for actin polymerization. However, further study in this limited explanation is required. Taken together, Nck1 expression in LEC might contribute to the recruitment of kinase proteins for PDGFR phosphorylation.

In fact, PDGFR triggering by PDGF-BB is likely to expose the phosphorylated site for Nck binding via Nck SH2 domain. This would be a crucial proximal event of PDGFR phosphorylation leading to downstream cellular outcomes. It has been shown that the PDGFRβ-Y751F mutant abolished Nck1 binding following PDGFR stimulation^21^. Apart from that, another phosphorylated site, Tyr-740, still functions as a harboring site for p85 subunit of phosphatidylinositol 3-kinase (PI3K) and its substrate Akt, was not impaired in PDGFRβ-Y751F mutant. This is contradictory to our result, where there is a dramatic decline of Akt phosphorylation in shNck1-LEC relative to the decreased PDGFR phosphorylation (Fig. 6D,E). It is possible that the Tyr-740 phosphorylation was diminished by the decreased PDGFR phosphorylation.

On the other hand, the failure of Nck1 binding to the PDGFR might disturb downstream intercellular signaling, which was associated with the reduction of Erk1/2, a member of mitogen activation protein kinase (MAPK) pathways, in shNck1 LEC (Fig. 6F,G). These results agreed with a previous work, in which PDGF is involved in cell migration of LEC via PI3K/Akt and MAPK after PDGFRβ engagement^32^. Based on the obtained results, it is apparent that the Nck1 adaptor protein might participate in transducing signals from receptor tyrosine kinases, PDGFRs, to downstream signaling pathways through the PI3K/Akt and MAPK signaling pathways.

**Figure 7:**
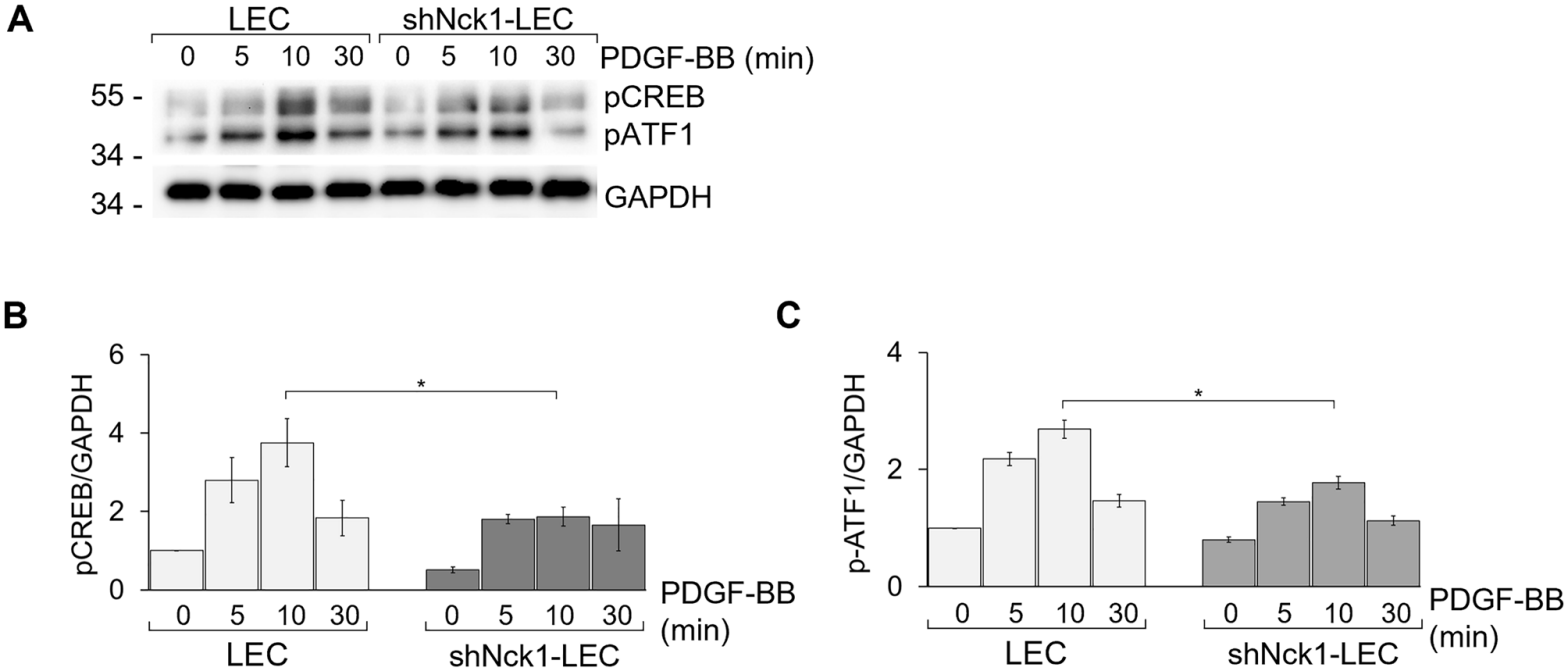
Nck1 knockdown disturb the phosphorylation of CREB and ATF1 LEC and shNck1 LEC were either stimulated with 30 ng/ml PDGF-BB or left untreated at 37 °C for the indicated times. Cell lysates were separated by SDS-PAGE and the western blot was probed with (**A**, **B**) anti-phospho-CREB and (**A**, **C**) anti-phospho-ATF1 antibodies. The quantified signal intensities are presented as a ratio of the protein of choice to the corresponding GAPDH values normalized to the value of unstimulated LEC. Data are representative of three experiments (mean ± SEM). *p < 0.05.

Notably, the activation of cell survival-related transcription factors, CREB and activating transcription factor 1 (ATF1), mediates cell proliferation through PI3K and MAPK pathways and suppression of both kinase proteins would disturb the cell survival and cell development^51,52^. CREB is a vital transcription factor participating in the regulation of cell cycle progression^52–54^. Our result showed that there was a dramatic increase of the CREB and ATF-1 phosphorylation in stimulated LEC. In contrast, phosphorylation of CREB and ATF1 was sharply declined in shNck1-LEC (Fig. 7A–C). In line with this finding, reduced Nck1 expression impaired the PDGFR-dependent signaling, resulting in LEC arrest in the G0/G1 phase. One possible influence on the outcomes might be from the attenuation of CREB and ATF1 phosphorylation as we found in this work. Together, these findings showed that Nck1 might regulate cell cycle progression via CREB-ATF1 activation.

## Conclusion

In sum, our present study highlights the functional role of the Nck1 adaptor protein in lens epithelial cells. Knockdown of Nck1 protein suppressed PDGF-BB-stimulated cell proliferation, migration, and actin polymerization by disturbing Erk1/2, Akt, CREB and ATF1 signaling. These findings suggested that the Nck1 adaptor protein may be a potential therapeutic protein target for treating lens epithelial cell growth-related ocular disorders.

## Methods

### Reagents and antibodies

In this work, the following antibodies were used. Rabbit anti-Nck1, phospho-AKT (S473), phospho-ERK1/2 (T202/T204), phospho-CREB (S133) and GAPDH antibodies were purchased from Cell Signaling (Danvers, MA), Rabbit anti-PDGFRB antibody was from Thermo Fisher Scientific (USA). Recombinant Human Platelet-Derived Growth Factor-BB (PDGF-BB) was purchased from Gibco (Waltham, MA) and Phalloidin CruzFluor™ 488 Conjugate for F‐actin staining was purchased from Santa Cruz Biotechnology (Santa Cruz, CA, USA).

### Cell culture

Human lens epithelial cell line (LEC, clone B3) was obtained from ATCC (CRL-11421) and cultured in Minimum Essential Medium Eagle medium (MEM) (Gibco) supplemented with 10% heat-inactivated fetal bovine serum (Gibco) and 100 U/ml penicillin and 100 µg streptomycin (JRH Biosciences) at 37 °C in the humidified atmosphere with 5% CO_2_.

### Generation of Nck1 knockdown LEC (shNck1 LEC)

In order to investigate Nck1 protein functions in LEC, the pLVX-shRNA vector encoding an Nck1-specific short hairpin RNA (pLVX-shNck1) (Takara Shuzo, Tokyo, Japan) was constructed as previously described^26^. The shRNA target sequence against Nck1 was as follows: 5′-GGGTTCTCTGTCAGAGAAA-3′^26^. The downregulation of Nck1 adaptor protein in LEC was achieved using pLVX-shNck1 transfection. Transfection of the constructed vector into LEC was carried out with an electroporator using Amaxa Nucleofection II (Lonza). Cells were transfected with the Nck1-shRNA expressing vectors, pLVX-shNck1. A stable clone of Nck1 knockdown LEC was then obtained by culturing the transfected cells in media supplemented with puromycin followed by limiting dilution. The efficacy of Nck1 protein reduction and PDGFR expression was assessed by western blotting analysis.

### Cell proliferation assay

For cell proliferation measurement, a 5-bromo-2′-deoxyuridine (BrdU) incorporated-DNA content of actively proliferating cells was detected using a colorimetric BrdU (5-bromo-2′-deoxyuridine) ELISA kit (Roche Diagnostics, Mannheim, Germany). A Cell density at 5 × 10^3^ cells per well were seeded into 96-well culture plates in the presence or absence of 30 ng/ml of PDGF-BB for 24 and 48 h. Cells were additionally incubated for 24 h with 20 μl/well of BrdU labeling reagent according to the manufacturer's instructions, resulting in BrdU-labeled proliferating cells. Absorbance was measured at 450 nm on a microplate reader (Perkin Elmer, MA, USA). All proliferation assays were performed in independent triplicates. The quantity of BrdU incorporated into cells was calculated in proportion to the absorbance of the developed color, indicating cell proliferation.

### Scratch wound healing assay

For the wound-healing assay, a scratch of the confluent cell monolayer was performed using sterile 200 μl tips. The reference of scratched lines can be made by drawing the dish with an ultrafine tip marker. Pictures of the baseline of the scratch area were taken under the microscope. The wound area was monitored following 24 h incubation. The covered wound area quantification was acquired using the ImageJ software.

### Cell cycle analysis

Cell cycle analysis based on DNA content was determined with flow cytometry. Cells were fixed in 70% cold ethanol overnight. Fixed cells were harvested and were then stained with propidium iodide (PI) in PBS containing 50 μg/ml RNase A for 30 min. Cells were analyzed on a FACS Calibur flow cytometer (BD Biosciences). Stained cells were analyzed for cell cycle distribution on a FACSCalibur (Becton Dickinson, NJ, USA) and data were analyzed with CellQuestPro software.

### Immunofluorescence visualization

For the actin cytoskeleton staining, cells were fixed with 4% paraformaldehyde, permeabilized with 0.1% Triton X-100 and blocked non-specific bindings with 2% fetal calf serum in PBS. After washing, actin filaments were stained with AlexFluor 488‐phalloidin staining. The stained cells of each experiment were mounted with a mounting solution with DAPI. Green fluorescence signals were observed under a 60× objective lens of a fluorescence microscope (Nikon Eclipse Ti, Nikon^®^, Melville, NY) using NIS‐Elements D software with 2560 × 1920 record pixels. Cell area, cell circularity, and stress fiber formation were quantified using ImageJ software.

### Cell stimulation, cell lysis and western blotting

For PDGF-BB stimulation, cells were harvested and starved in medium with 1% fetal bovine serum for 1 h at 37 °C. Subsequently, cells were either stimulated with 30 ng/ml of PDGF-BB for indicated time points or left untreated. In order to collect protein lysates, cells were then lysed in 100 µl lysis buffer (20 mM Tris–HCl (pH 8), 137 mM NaCl, 2 mM EDTA, 10% glycerol, phosphatase/protease inhibitor cocktail (Sigma), 1 mM PMSF, 5 mM iodoacetamide, 0.5 mM sodium orthovanadate, 1 mM NaF and 0.3% Brij96V) for 30 min on ice^26^. Then, cell lysates were subjected to SDS-PAGE and western blotting with the desired antibodies. The PVDF membrane was cut prior to antibody hybridization according to the protein size based on the protein band of interest, and visualization was done using a CCD camera (ImageQuant LAS 4000; GE Healthcare Life Sciences). The quantification of the band intensities was assessed by the ImageJ software (version 1.46r, https://imagej.nih.gov/ij/download.html). Uncropped gel images are provided in the “Supplementary information file”.

### Statistical analysis

Data are represented as mean ± standard errors of the mean (SEM). All differences between experimental groups were analyzed with the student’s t-test. Significant differences were considered when the p values were less than 0.05. (n.s. = not significant, *p < 0.05, **p < 0.01, ***p < 0.001).

## Supplementary Information

Supplementary Information.
