# Supplementary material for: Essential function of adaptor protein Nck1 in platelet-derived growth factor receptor signaling in human lens epithelial cells
Source: Sci Rep. 2022 Jan 20;12:1063. doi: 10.1038/s41598-022-05183-1 (PMC8776929; doi:10.1038/s41598-022-05183-1)

**Supplementary File**

Full-length membrane blots used in all figures are shown. Blots were imaged using a CCD camera (ImageQuant LAS4000, GE Healthcare Life Sciences) with a chemiluminescence channel. Molecular weights are given in kDa. M; marker lane.

For Figure1A

Nck1 and GAPDH – replicate 1


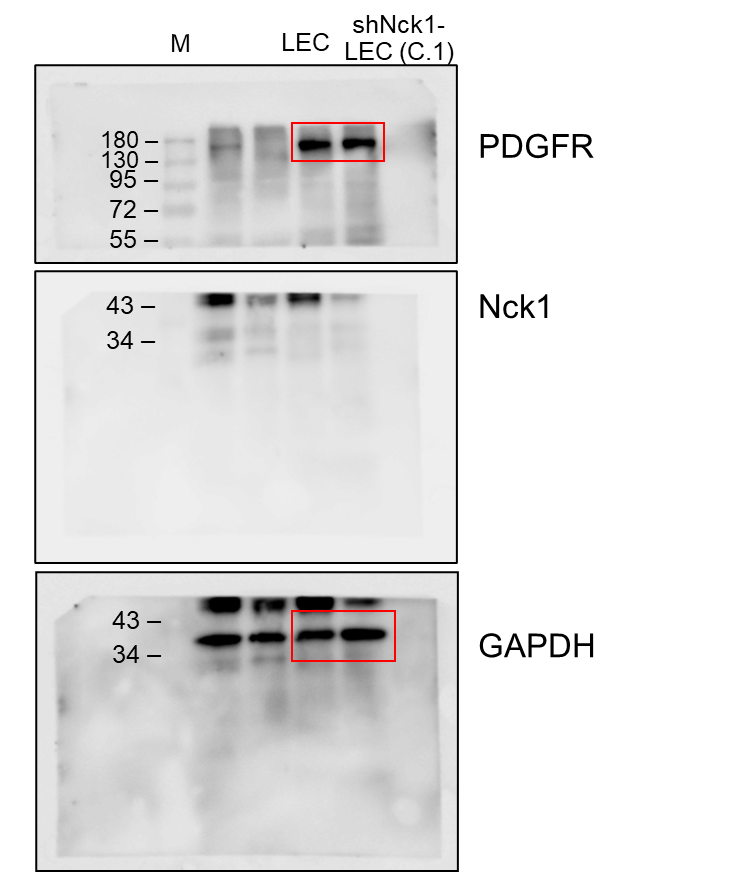


Nck1 and GAPDH – replicate 2


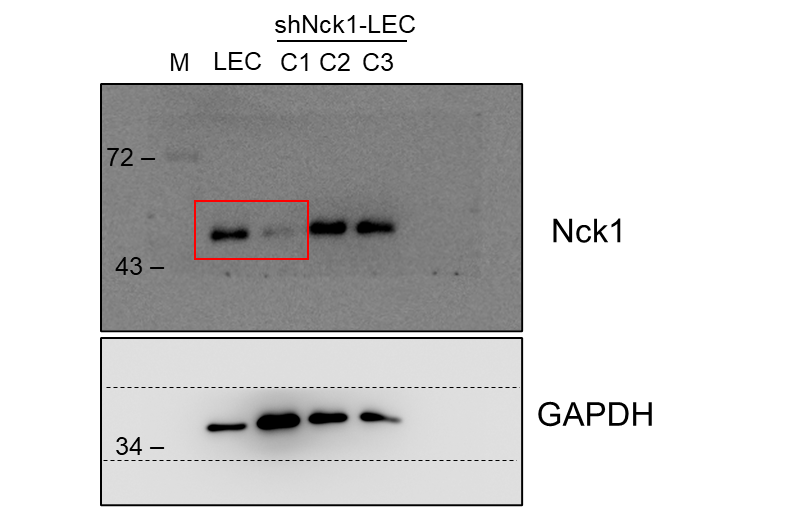


Nck1 and GAPDH – replicate 3


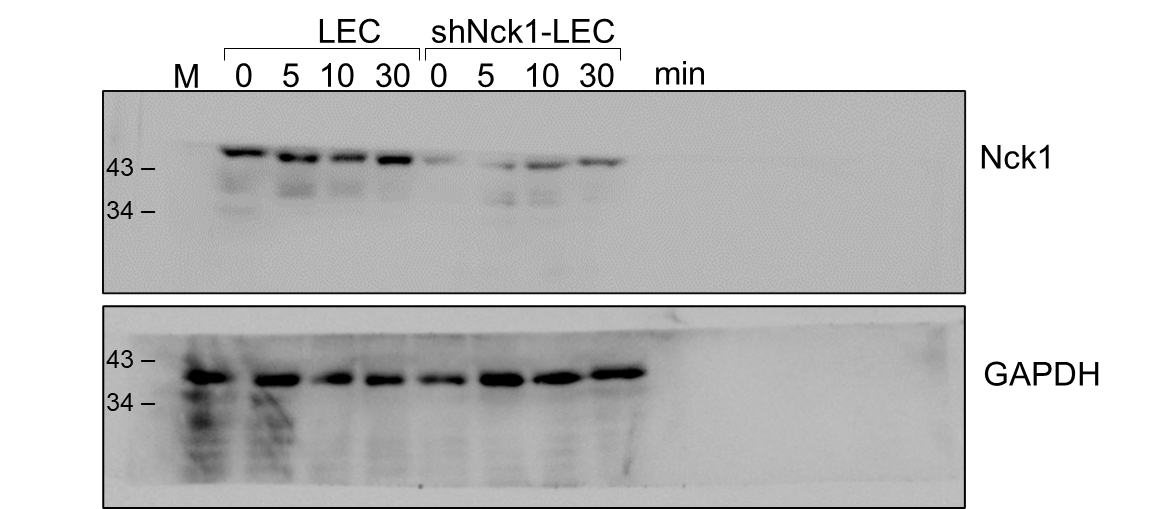


For Figure 6A

4G10– replicate 1


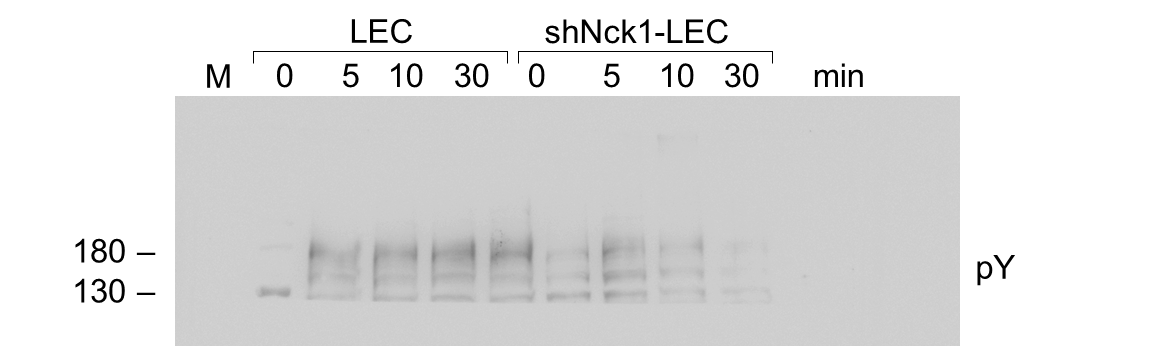


4G10 – replicate 2


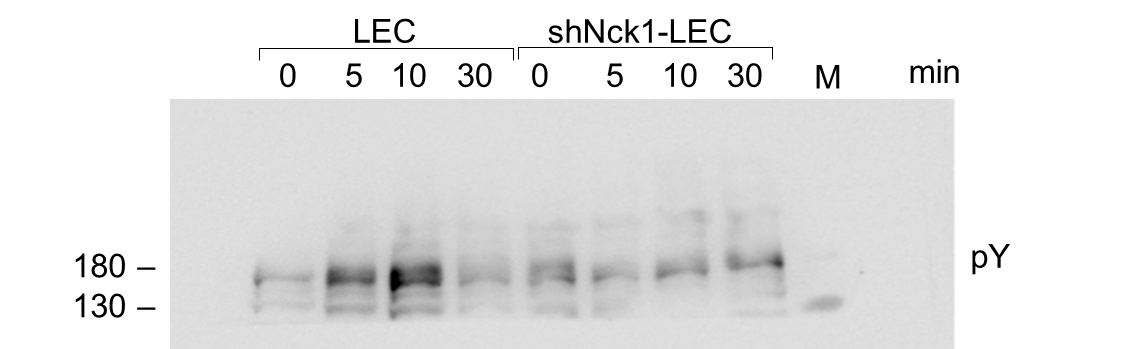


4G10 – replicate 3


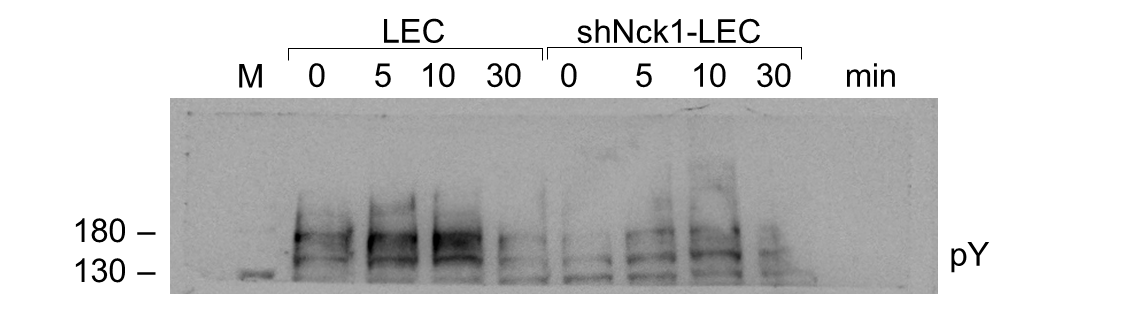


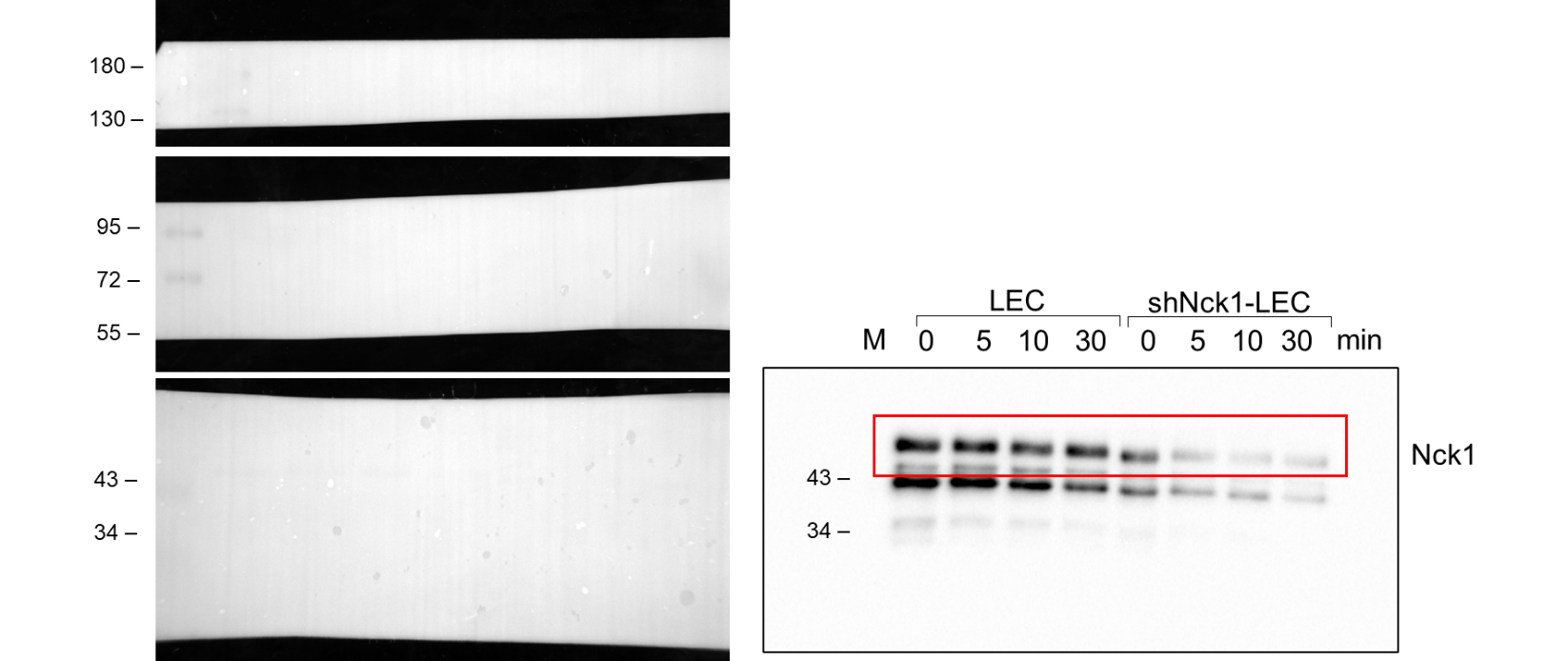
Nck 1 and actin – replicate 1


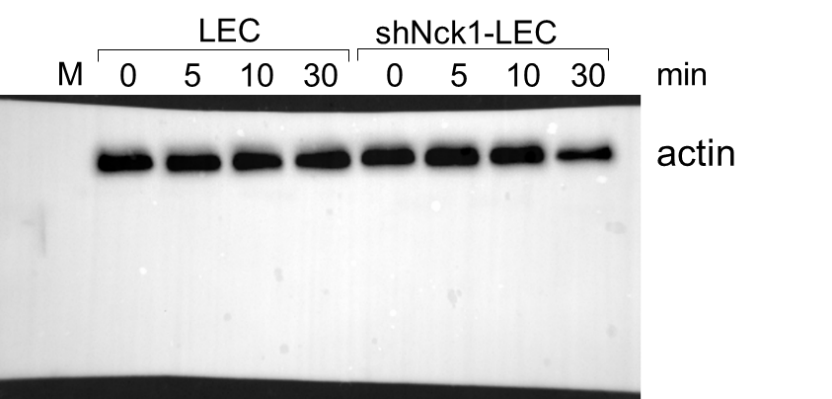


Nck 1 and GAPDH – replicate 2


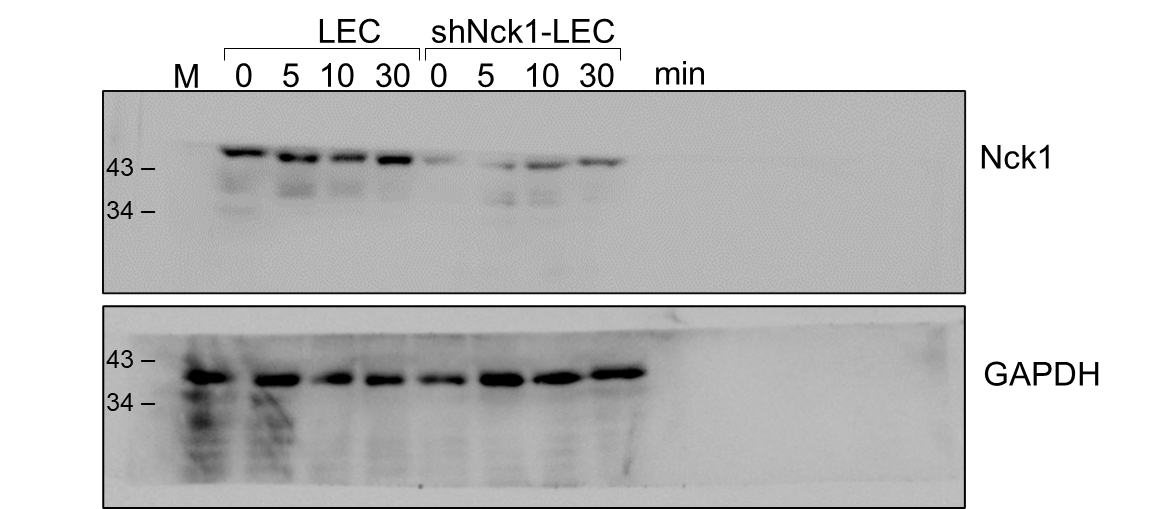


Nck 1 and actin – replicate 3


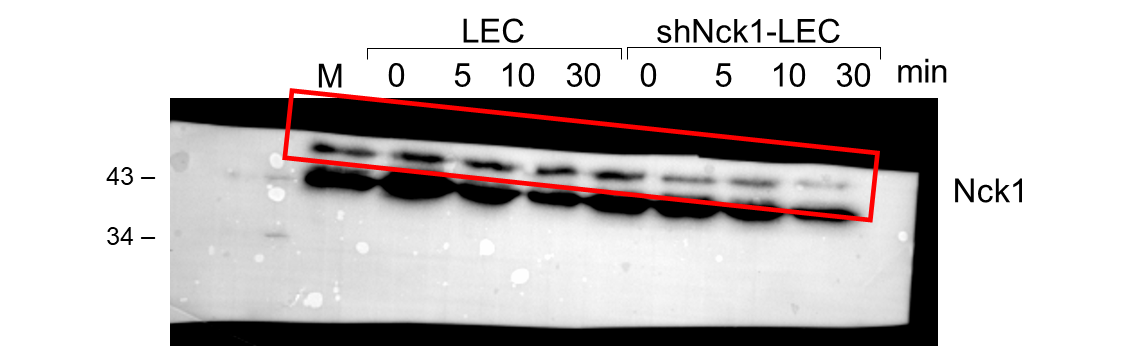


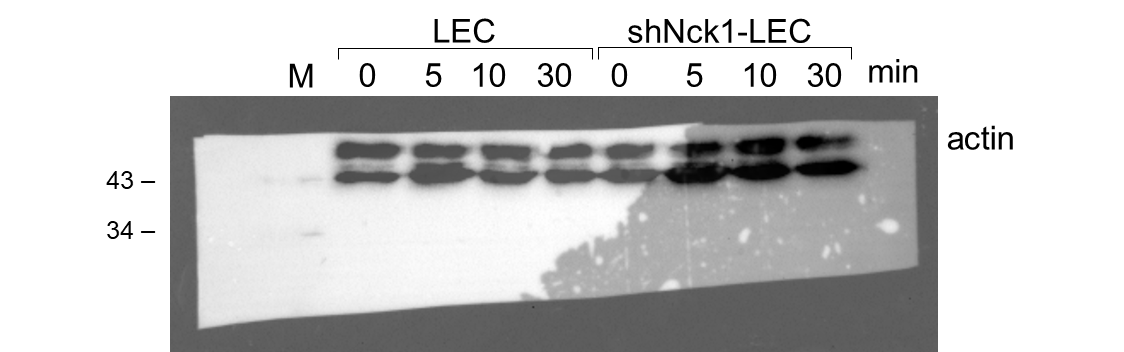


For Figure 6D

pAkt and total Akt – replicate 1


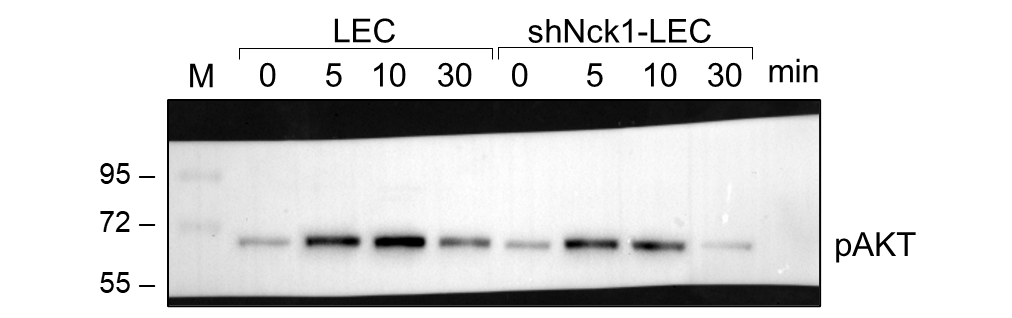


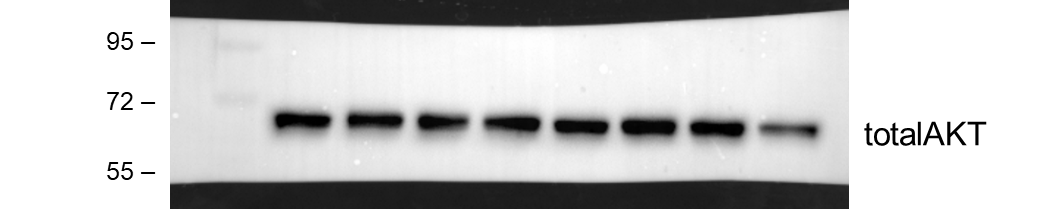


pAkt and total Akt – replicate 2


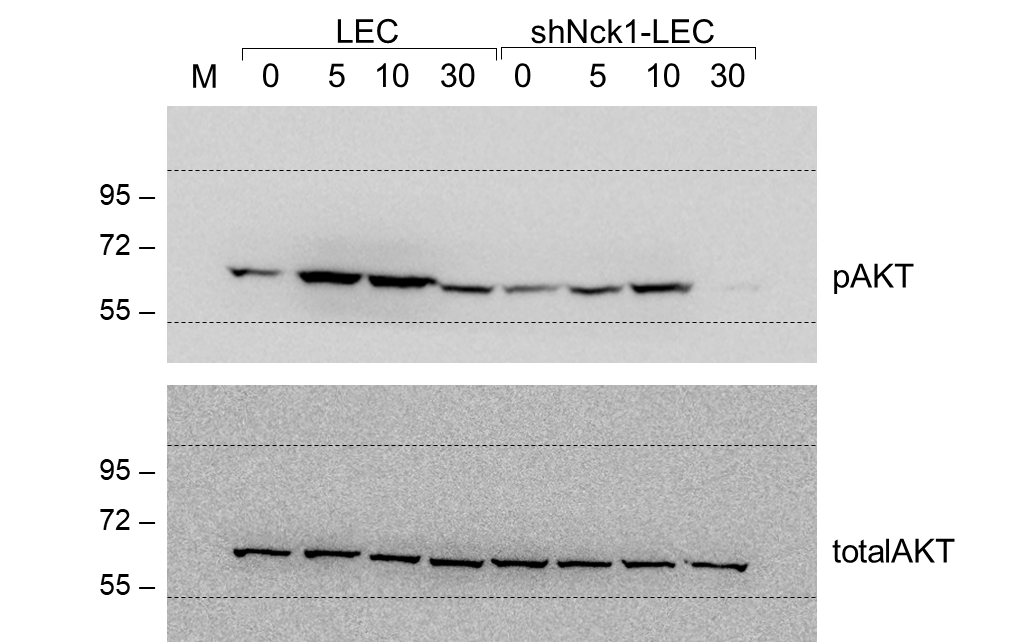


pAkt and total Akt – replicate 3


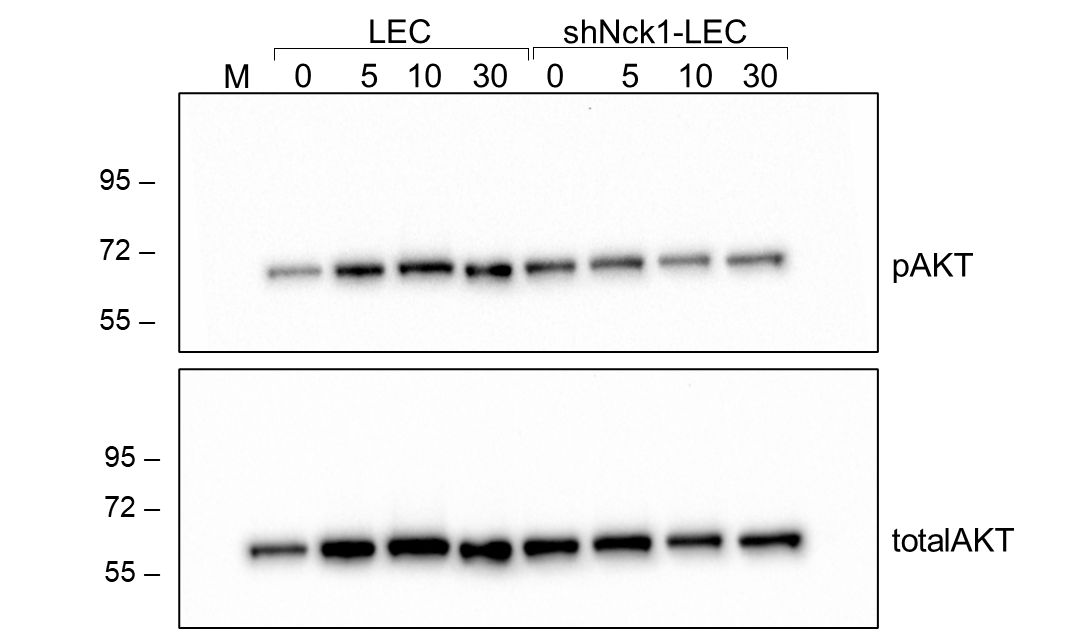


For Figure 6F

pErk and GAPDH – replicate 1


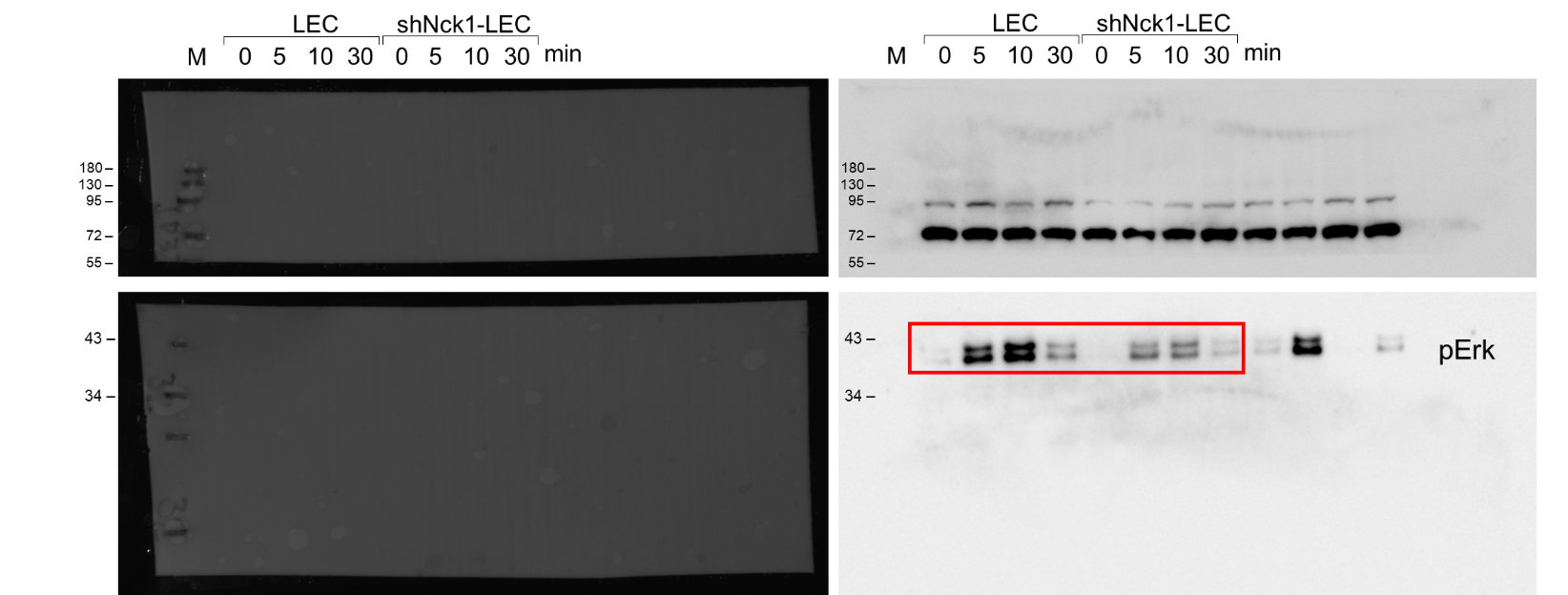


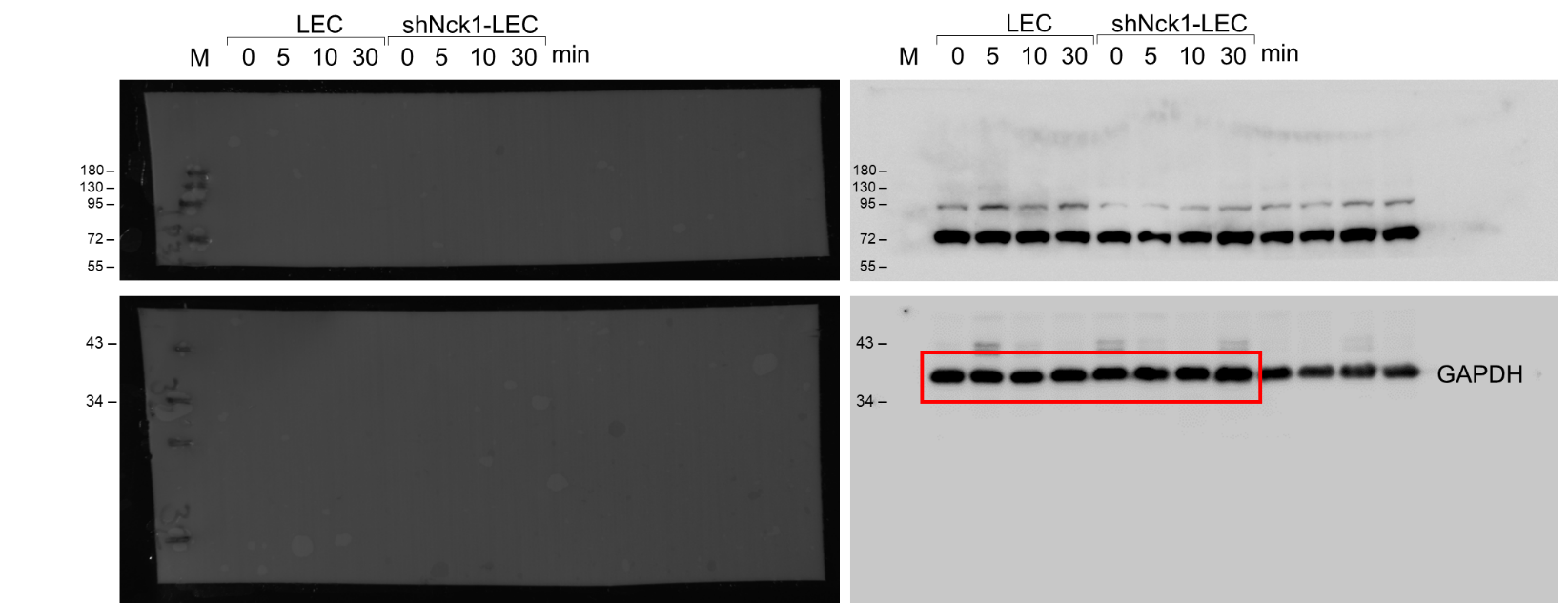


pErk and GAPDH – replicate 2


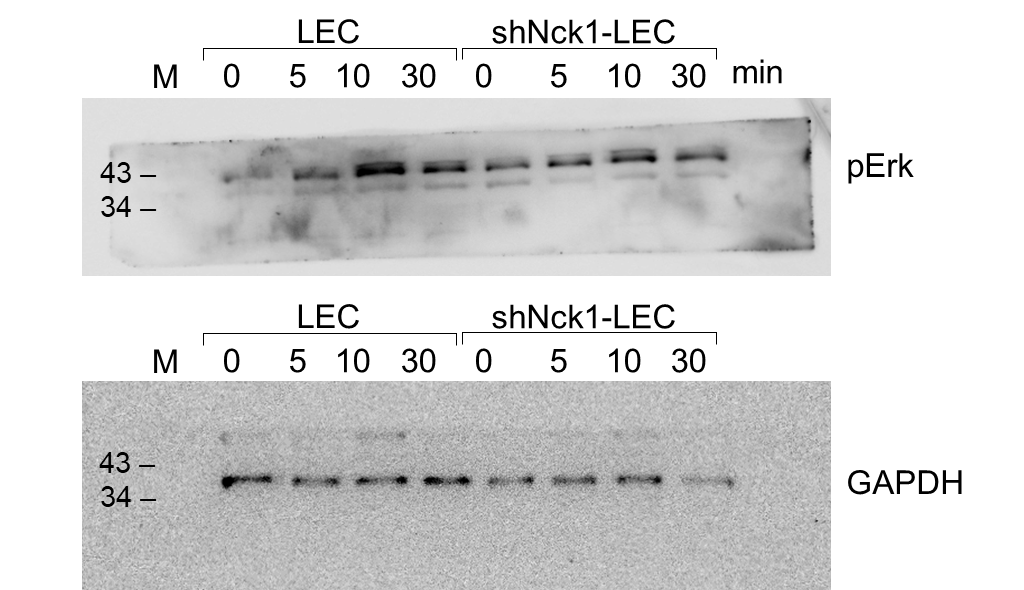


pErk and actin – replicate 3


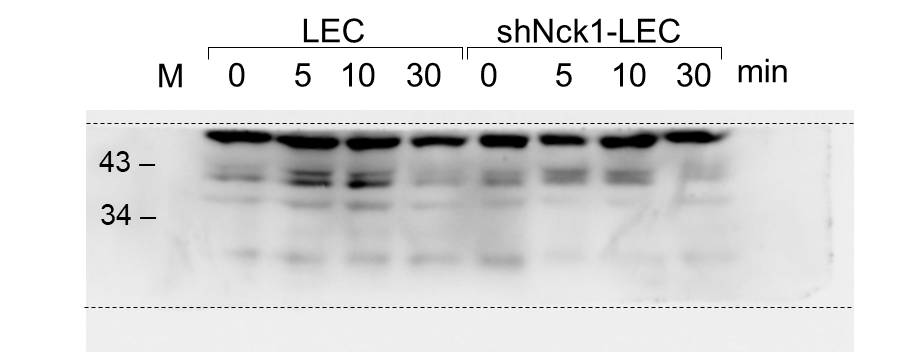


For Figure 7A

pCREB and actin – replicate 1


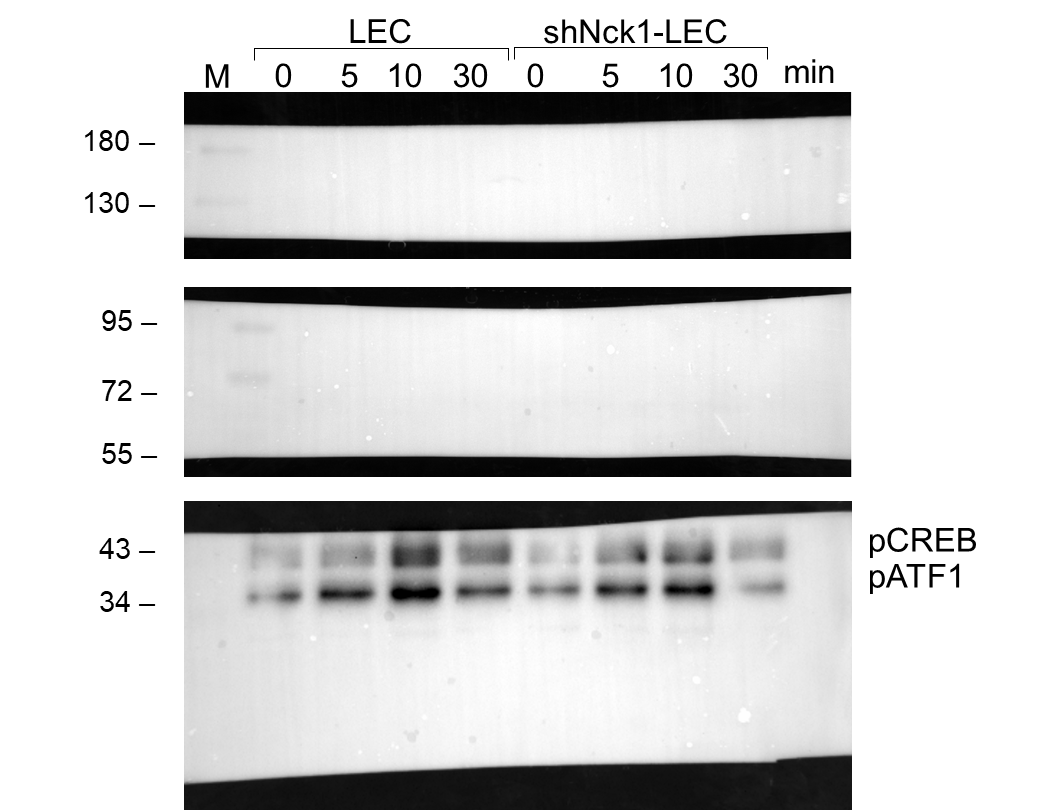


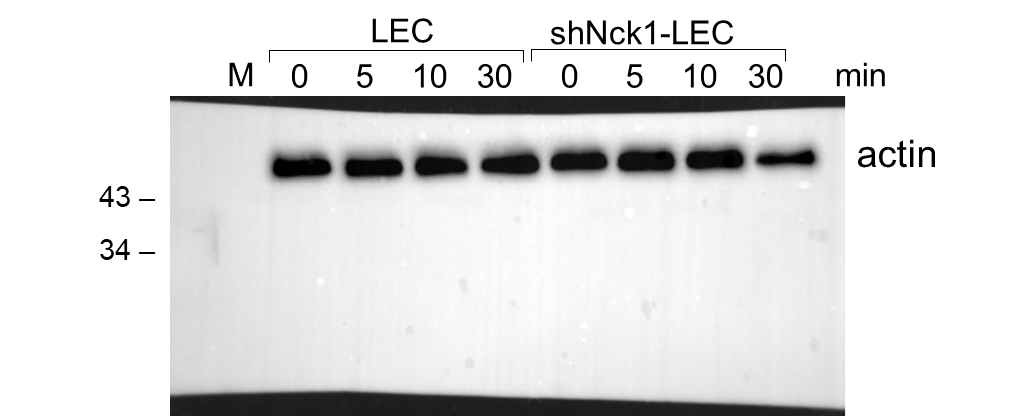


pCREB and GAPDH – replicate 2


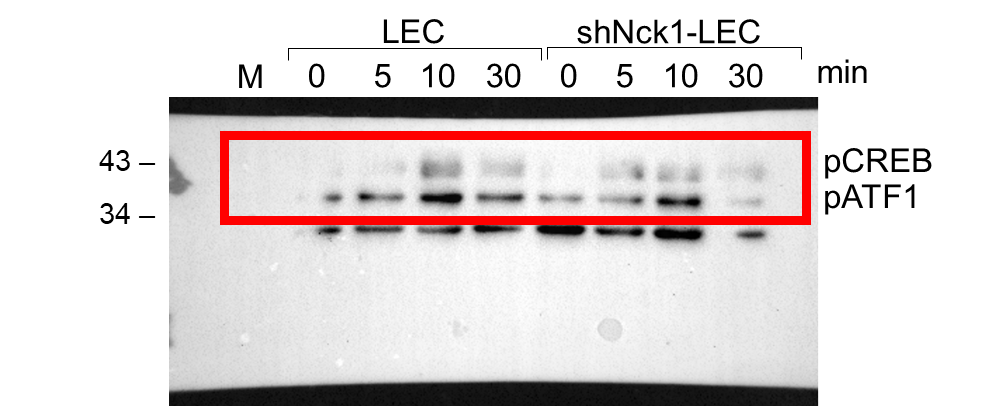


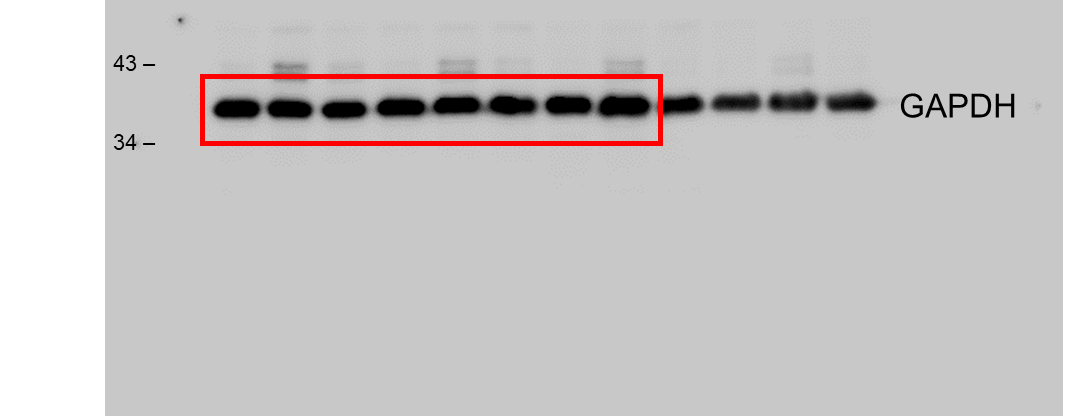


pCREB and GAPDH – replicate 3


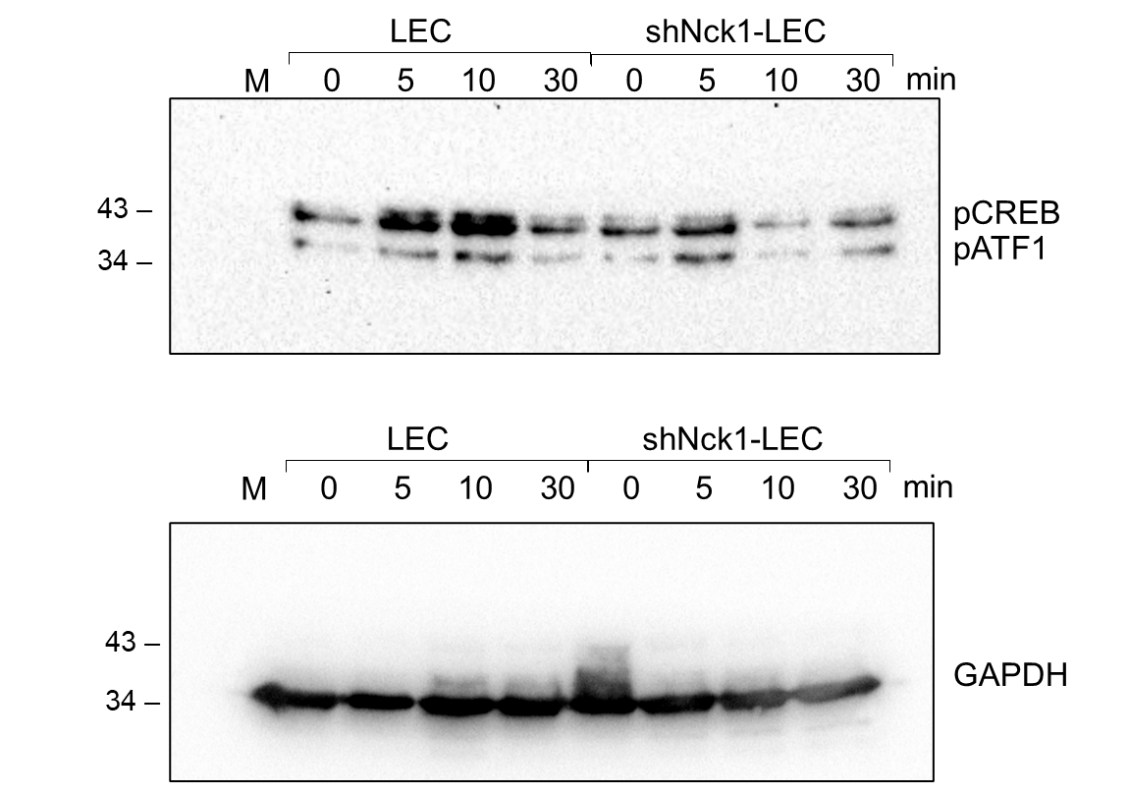

Supplement: Supplementary file 1 — Supplementary Information. [file 41598_2022_5183_MOESM1_ESM.docx]
